# Acidification of rat TRPV1 alters the kinetics of capsaicin responses

**DOI:** 10.1186/1744-8069-1-28

**Published:** 2005-09-28

**Authors:** Torben R Neelands, Michael F Jarvis, Ping Han, Connie R Faltynek, Carol S Surowy

**Affiliations:** 1 Neuroscience Research, Global Pharmaceutical Research and Development, Abbott Laboratories, R04PM, AP9A, Abbott Park, IL, 60064-6123, USA

**Keywords:** kinetics, electrophysiology, patch-clamp, pharmacology, TRPV1, acid, capsaicin

## Abstract

TRPV1 (vanilloid receptor 1) receptors are activated by a variety of ligands such as capsaicin, as well as by acidic conditions and temperatures above 42°C. These activators can enhance the potency of one another, shifting the activation curve for TRPV1 to the left. In this study, for example, we observed an approximately 10-fold shift in the capsaicin EC_50 _(640 nM to 45 nM) for rat TRPV1 receptors expressed in HEK-293 cells when the pH was lowered from 7.4 to 5.5. To investigate potential causes for this shift in capsaicin potency, the rates of current activation and deactivation of whole-cell currents were measured in individual cells exposed to treatments of pH 5.5, 1 μM capsaicin or in combination. Acidic pH was found to both increase the activation rate and decrease the deactivation rate of capsaicin-activated currents providing a possible mechanism for the enhanced potency of capsaicin under acidic conditions. Utilizing a paired-pulse protocol, acidic pH slowed the capsaicin deactivation rate and was readily reversible. Moreover, the effect could occur under modestly acidic conditions (pH 6.5) that did not directly activate TRPV1. When TRPV1 was maximally activated by capsaicin and acidic pH, the apparent affinity of the novel and selective capsaicin-site competitive TRPV1 antagonist, A-425619, was reduced ~35 fold. This shift was overcome by reducing the capsaicin concentration co-applied with acidic pH. Since inflammation is associated with tissue acidosis, these findings enhance understanding of TRPV1 receptor responses in inflammatory pain where tissue acidosis is prevalent.

## Background

The vanilloid receptor 1 (TRPV1) is a member of the transient receptor potential family (TRP) of non-selective cation channels [1]. These receptors are activated by a variety of lipids, acidic conditions and temperatures above 42°C. TRPV1 channels are tetramers composed of subunits with six transmembrane spanning domains, a pore loop between TM5 and TM6, and large N- and C-terminal intracellular domains [2]. An intracellular domain just C-terminal to TM6 has been characterized as being important in the tetramerization of the channel and is coincident, in part, with the TRP box that is common among this family of ion channels [3].

The structural features of TRPV1 suggest that the primary ligand interaction site(s) and important regulatory mechanisms for the channel are intracellular. Indeed, multiple mutagenesis studies have shown that distinct intracellular regions are necessary for the binding of the exogenous TRPV1 agonist, capsaicin [4-6] although an extracellular site may also contribute to capsaicin binding [7]. In contrast, extracellular site acidic residues have been implicated in proton activation (at pH < 6) and sensitization of TRPV1 [8]. Further evidence that TRPV1 activation mechanisms are different for capsaicin and protons is provided by site-directed mutagenesis studies that disrupt capsaicin activation of the channel but leave proton actions intact [9]. Despite these differences, there is evidence of some commonality in the gating of the channel in response to capsaicin or acidic pH activation [10].

Under pathological conditions multiple agents may simultaneously influence the activity of TRPV1 receptors. For instance, inflammation, ischemia, and infections result in elevated proton concentrations that can reduce the pH below 6 in the surrounding tissues [11]. Acidic pH has been shown to stimulate a subpopulation of sensory nerves that are also activated by capsaicin [12]. In addition, disruption of the TRPV1 gene attenuates proton-induced excitation of C-fibers [13], supporting a key role for TRPV1 in inflammatory pain. Treatment of TRPV1 receptors with capsaicin in the presence of other activators, including heat and acid results in a leftward shift of the capsaicin concentration response curve [11,14]. This suggests additive or synergistic effects of acid or heat on TRPV1 activation by capsaicin. Such effects may occur through changes in capsaicin affinity or gating.

In this study, we found that acidic conditions (pH 6.5 to 4.0) alter both the activation and deactivation rate of capsaicin-activated currents, resulting in increased potency of capsaicin for TRPV1, with no change in efficacy. In contrast, the inhibitory potency of a novel competitive TRPV1 antagonist, A-425619, was significantly lowered when the two activators were co-applied. These results highlight the breadth of TRPV1 responses to different stimuli and the concept that this channel (as well as other TRP channels) may act not only as an integrator of different physical stimuli but also as a coincidence detector that may be important in determining the resultant physiological response to endogenous activators.

## Results

### Activation and inactivation kinetics of TRPV1 channels in response to capsaicin or acidic pH

**Figure 1:**
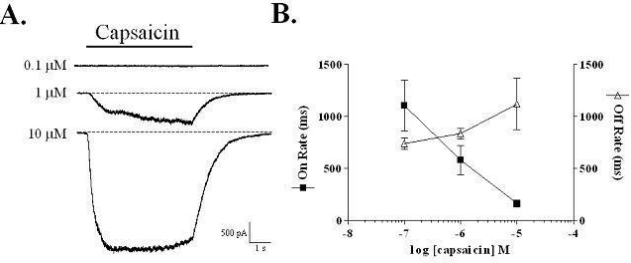
**Kinetics of capsaicin-activated currents in HEK293 cells stably expressing rat TRPV1 channels**. Application of increasing concentrations of capsaicin to cells voltage clamped at -60 mV result in progressively larger inward currents with faster activation rates and slower deactivation rates. (A) Representative current traces. (B) Plot of average activation and deactivation rates as a function of capsaicin concentration. Data are the mean ± SEM.

Whole-cell patch-clamp electrophysiological techniques were employed to characterize the responses of rat TRPV1, stably expressed in HEK-293 cells, to varying concentrations of capsaicin. Short (5 s) applications of capsaicin (0.1–10 μM) to rat TRPV1-expressing cells held at -60 mV resulted in a concentration-dependent increase of inward currents (Figure 1A). The rates of activation and deactivation of these responses were calculated using a single exponential equation (see methods). As capsaicin concentrations increased, the rate of activation dramatically increased from a τ of 1107 ± 242 ms at 0.1 μM to 582 ± 141 ms at 1 μM and 164 ± 33 ms at 10 μM (n = 4 – 6) (Figure 1B). In contrast, the deactivation rate slowed slightly with increasing concentrations of capsaicin (741 ± 56, 835 ± 54, 1121 ± 249 ms for 0.1, 1 and 10 μM capsaicin respectively; n = 4 – 6) (Figure 1B).

**Figure 2:**
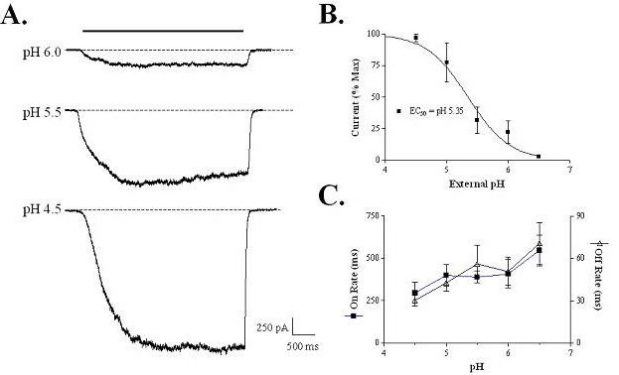
**Kinetics of acid-activated currents in HEK293 cells stably expressing rat TRPV1 channels**. Application of solutions with increasing proton concentrations to cells voltage-clamped at -60 mV result in progressively larger inward currents with slightly slower activation and deactivation rates. (a) Representative current traces. (b) pH response curve of average normalized currents. (c) Plot of average activation and deactivation rates as a function of pH. In contrast to the activation rates, which are similar between capsaicin and acid, the deactivation rates of acid responses are much faster than the capsaicin responses. Data are the mean ± SEM.

Similar experiments were performed to investigate the whole-cell current kinetics of rat TRPV1 in response to acidification of the extracellular solution. Decreasing the extracellular pH from 7.4 to 4.5 in stepwise fashion resulted in a pH dependent increase in inward current (Figure 2A). Average normalized peak currents were plotted as a function of the extracellular pH and fit with a logistic equation resulting in an EC_50 _of pH 5.35 and Hill slope of 1.3 (n = 6) (Figure 2B), which is similar to previous reports for acid activation of TRPV1 [11]. Activation rates increased from 546 ± 89 ms at pH 6.5 to 298 ± 62 ms at pH 4.5 (n = 3) (Figure 2C). A slight increase in the deactivation rate (70.5 ± 14.7 ms at pH 6.5 to 30.1 ± 3.7 ms at pH 4.5; n = 3 – 4) was found as pH was lowered (Figure 2C).

### Effect of acidic conditions on the potency of capsaicin for rat TRPV1

**Figure 3:**
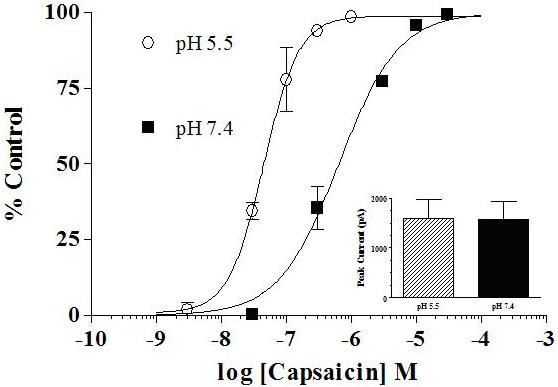
**Capsaicin concentration-dependent activation of rat TRPV1 is shifted to the left under acidic conditions**. Capsaicin potency is increased ~10 fold when the pH of the capsaicin-containing external solution is dropped from pH 7.4 (EC_50 _= 640 nM, closed squares) to pH 5.5 (45 nM, open circles). Currents evoked by application of pH 5.5 alone or that were required to hold the cell at -60 mV (resting holding current) were subtracted from peak responses. Data are the normalized average (mean ± SEM) of the resulting maximal currents for each condition. **Inset) **Peak responses of currents evoked by 30 μM capsaicin were compared at pH 7.4 and pH 5.5 on the same cell. No difference was detected in these experiments indicating that acidic conditions did not alter the efficacy of capsaicin. Data are the mean ± SEM.

Capsaicin responses were measured on the same cell at pH 7.4 (30 nM – 30 μM) and pH 5.5 (3 nM – 1 μM) to determine if there was a similar shift in the potency of capsaicin. Peak currents were normalized to the maximum response for each condition and the averages were plotted as a function of capsaicin concentration (n = 6–7). The resulting dose response curves illustrate a significant leftward shift in the EC_50 _for capsaicin from 640 nM (n_H _= 0.9) at pH 7.4 to 45 nM (n_H _= 1.6) at pH 5.5 (Figure 3). Peak inward currents evoked by application of 30 μM were not significantly different at pH 7.4 (1577 ± 349 pA) than at pH 5.5 (1596 ± 366 pA) (n = 7), indicating that there was no change in efficacy at this concentration of capsaicin (Figure 3, inset).

### The effect of acidic pH on the activation rate of capsaicin-evoked currents

**Figure 4:**
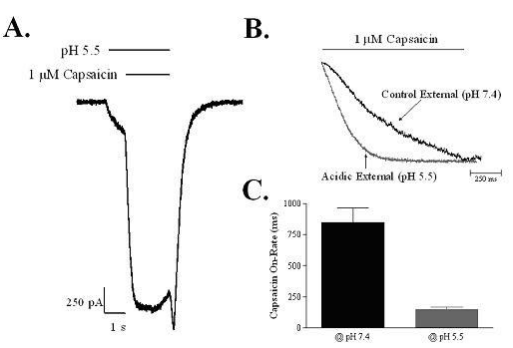
**Acidic pH increases the activation rate of capsaicin-activated responses of rat TRPV1 channels expressed in HEK-293 cells**. Comparison of the activation rate of 1 μM capsaicin-activated currents in rat TRPV1 expressing cells under control conditions (pH 7.4) and after pre-application of acidic solution (pH 5.5). (A) Representative current trace. (B) Overlay of normalized current traces illustrating increased activation rate of capsaicin under acidic conditions. (C) The activation rate of capsaicin-activated currents was significantly faster at pH 5.5 (149 ± 17 ms) than at pH 7.4 (847 ± 119 ms) (n = 4). Data are the mean ± SEM.

The mechanism by which acidic pH increases the potency of capsaicin at the rat TRPV1 receptor was studied by co-applying the two activators either simultaneously or in a variety of sequences. Activation and deactivation rates of the resulting currents were measured. In the first set of experiments, the channel was exposed to pH 5.5 alone for 1 second, followed by 3 seconds of co-application with 1 μM capsaicin (Figure 4A). This protocol resulted in capsaicin-activated currents with much faster activation rates than occurred with the same capsaicin concentration at pH 7.4 (Figure 4B). The average activation rate was significantly increased from 847 ± 119 ms at pH 7.4 to only 149 ± 17 ms at pH 5.5 (n = 4) (Figure 4C). Moreover, the activation rate of the response to pH 5.5 increased after prior application of 1 μM capsaicin (τ = 93 ± 13 ms) compared with conditions where only pH 5.5 solutions were applied (τ = 917 ± 307 ms; n = 3), suggesting that capsaicin may increase the affinity of protons to rat TRPV1 (data not shown).

It has previously been reported that mildly acidic pH, which alone does not cause activation of TRPV1, produces changes in the response to capsaicin [11]. Therefore, we varied the pH of the pre-pulse to determine the effect of pH on the activation rate of capsaicin-evoked currents. Reducing the pH to 6.8 did not directly activate TRPV1 channels (Figure 2) but resulted in a subtle increase in the activation rate of capsaicin responses (Table 1). Increasing the acidity of the solution to pH 6.0 or pH 5.0 resulted in further significant increases of the activation rate of capsaicin currents (Table 1).

**Table 1:** Acid dependent effects on the activation rate of capsaicin-activated responses. Increasing the acidity of the external solution during a 1 second pre-pulse resulted in an increase in the activation rate of capsaicin-evoked currents in rat TRPV1 expressing HEK-293 cells. Values are the mean ± SEM of the calculated activation rate (τ) using a single exponential fit.

**Condition**	**τ (ms)**	**n**
**Control (pH 7.4)**	1176 ± 195	15
**pH 6.8**	1021 ± 128	15
**pH 6.0**	632.0 ± 86.5	13
**pH 5.0**	242.7 ± 49.7	7

### The effect of acidic pH on the deactivation rate of capsaicin-evoked currents

**Figure 5:**
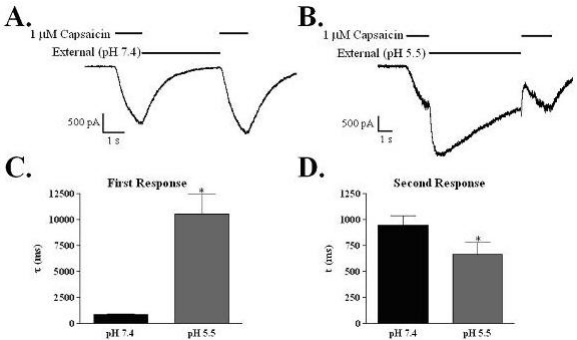
**Acidic pH decreases the deactivation rate of capsaicin-activated responses of rat TRPV1 channels expressed in HEK-293 cells**. A double application protocol was used to investigate the effect of different pH's on the off rate of capsaicin. Two short applications of capsaicin (1 s) were interrupted by 1 to 5 second applications of either control external solution (pH 7.4) or acidic external (pH 5.5). A). Representative trace illustrating that the deactivation rates of both capsaicin applications are similar when the interpulse interval is at physiological pH. B) In contrast, when the interpulse interval is under acidic (pH 5.5) conditions, the deactivation rate of the initial application is substantially slower as illustrated by a representative trace. C) Comparison of the effect of external pH on the deactivation rates following the first capsaicin application. D) Comparison of the effect of external pH on the deactivation of the second capsaicin application. Data are the mean off rates (τ) ± SEM.

The effect of acidic pH on the deactivation rate of capsaicin currents was tested using a paired-pulse protocol. In these experiments two short (1 s) applications of capsaicin were separated by 1–5 seconds of external solution at either pH 7.4 or pH 5.5. The 5 s interruptions are shown in Figure 5; similar results were obtained at shorter interpulse intervals. The deactivation rates of the first capsaicin responses were much slower with an acidic extracellular solution (τ = 10553 ± 1880 ms) (Figure 5B,C) than with an extracellular solution at pH 7.4 (τ = 854 ± 60 ms) (Figure 5A,C). In contrast, the deactivation rates in response to the second capsaicin application, reflecting the return to external solution of pH 7.4 in both cases, were only slightly faster following acid exposure than when the interpulse solution remained at physiological pH (τ = 944 ± 94 ms vs. 665 ± 114 ms; n = 4) (Figure 5D). These results suggest that acidic pH has a limited time-dependent effect on the channel. These effects of capsaicin, pH 5.5 or a combination of the two, on the activation and deactivation rates of evoked currents are summarized in Table 2.

**Table 2:** Comparison of the activation and deactivation kinetics of TRPV1. Mean activation and deactivation rates of responses from TRPV1 expressing HEK-293 cells following application of capsaicin (1 μM), acidic pH (5.5) or the combination. Row 3 represents activation and deactivation rates of currents evoked by 1 μM capsaicin following pre-exposure to acidic conditions (pH 5.5). Row 4 represents the activation rates of acid-evoked currents following pre-exposure to capsaicin (1 μM). The absolute value of rate measurements had slight day-to-day variations (possibly due to relative position of the drug application tube). Therefore values in this table are from cells in which direct comparisons can be made. Values are mean ± S.E.M.

	**Activation Rates**		**Deactivation Rates**	
	
	τ (ms)	n	τ (ms)	n
**1 μM Capsaicin**	847 ± 119	4	845 ± 60	4
**pH 5.5**	917 ± 307	3	56 ± 13	4
**1 μM CAP (pH 5.5)**	149.3 ± 17	4	10553 ± 1880	4
**pH 5.5 (1 μM CAP)**	93 ± 13	3	-not tested-	

Note: Rates from experiments shown in Figures 4 and 5.

**Figure 6:**
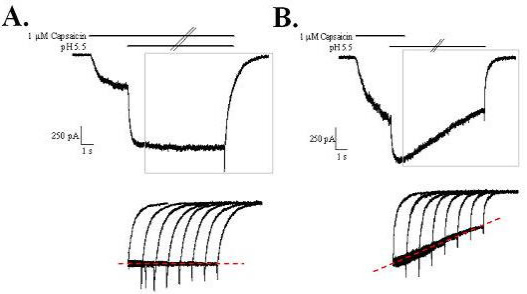
**Prolonged exposure of TRPV1 to acidic pH produces a time-dependent change in the deactivation rate of capsaicin-activated currents**. Single pulse protocols were used to determine time dependent differences in the deactivation of capsaicin currents following prolonged exposure to acidic pH. A) Co-application of acid (pH 5.5) and capsaicin produced sustained currents independent of the duration of application. TRPV1 channels were activated by 1 μM capsaicin for 4 s, followed by 8 seconds of capsaicin + pH 5.5. The time dependence (1–8 s) of the sustained current is shown below and illustrates the reproducibility of the responses, the lack of desensitization and the parallel deactivation time-courses. B) Deactivation time constant of capsaicin response is decreased if acidic conditions persist after capsaicin application is terminated. Note the slow decline in current amplitude following removal of capsaicin, and the relatively faster deactivation rates of deactivation with longer intervals of acid pH application, presumably due to fast deactivating acid responses (see figure 2) contributing a greater proportion of the total current.

Additional experiments were performed to investigate the pronounced effect of acidic pH on the deactivation rate of capsaicin. A single application protocol was established in which TRPV1 channels were initially activated by 1 μM capsaicin followed by 1–8 seconds treatment with either pH 5.5 + capsaicin or pH 5.5 alone (Figure 6). When capsaicin was present throughout the protocol, the current amplitude showed little desensitization and was independent of the duration of application (Figure 6A). The deactivation phase following the termination of the co-agonist treatment had a biphasic time course with similar kinetics even as the duration of application was increased (bottom of Figure 6A). These characteristics were altered when capsaicin application was terminated but the acidic pH was maintained to the end of the protocol (Figure 6B). Under these conditions, the amplitude of the current declined in a linear fashion (dotted line, bottom Figure 6B) that was faster than when capsaicin was present but slower than when the external solution was at pH 7.4 (see Figures 1A and 5A). In addition, when the pulse duration was lengthened the biphasic current deactivation was comprised of primarily the fast deactivation component (bottom of Figure 6B).

**Figure 7:**
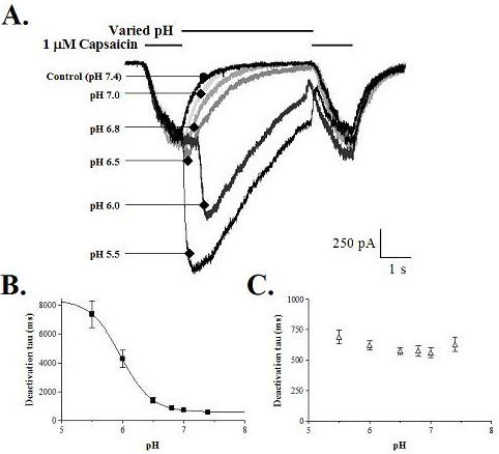
**Proton concentration dependent slowing of the capsaicin deactivation rate**. Two applications of capsaicin were interrupted by 5 s applications of external solutions with increasing proton concentrations. A) Representative current traces. The deactivation rate was slowed slightly by mildly acidic solutions that did not activate the channel directly but that potentiated the capsaicin response (pH 7.0 and 6.8). The slowing of the capsaicin deactivation rate was enhanced even more by application of acid that could directly activate the channel (pH 6.0 and 5.5). B) Concentration-dependent effects of pH on the deactivation-time constant of capsaicin. C) No concentration dependent effects were recorded for the deactivation rate of the second capsaicin application, indicating that previous exposure to acidic conditions does not affect subsequent capsaicin deactivation.

The pH dependent effects on the slowing of the capsaicin response were tested utilizing a paired-pulse protocol in which the pH of the solution was varied during the intervening segment (Figure 7). As illustrated in Figure 7A, lowering the pH to only 7.0 caused a small slowing of the off rate of the first pulse even though there was no significant change in the peak current (light gray line). The extent to which the deactivation rate was slowed was dependent on the acidity of the solution and was the greatest under conditions that produced direct channel activation (pH 6.5 and below – see figure 2B). Similar to the data shown in Figure 5B, the deactivation of the second capsaicin application was not affected by previous exposure to acid. The deactivation time of the first and second capsaicin applications was measured and the average rate was plotted as a function of the intervening pH (Figure 7B,C). The deactivation of the first capsaicin application slowed from a τ of 585 ± 43 ms at pH 7.4 to 7384 ± 929 ms at pH 5.5 (n = 7 – 8), whereas the deactivation of the second application ranged from 562 ± 40 to 689 ± 58 (n = 7 – 8) and showed no acid-dependent effects.

**Figure 8:**
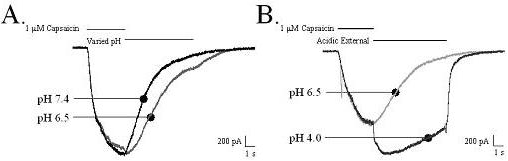
**A proton concentration unable to directly activate TRPV1 channels slows the deactivation rate of capsaicin responses**. Representative traces from two different cells stimulated by a single capsaicin pulse application that further illustrates the proton concentration dependent effects on capsaicin deactivation rates. A) Representative current traces showing that the deactivation of capsaicin-activated currents is slowed when the pH of the external solution was reduced (6.5) even though this pH did not significantly potentiate the capsaicin response. B) Representative current traces illustrating that lowering the pH to more acidic conditions results in both an increase in maximal current amplitude and a striking slowing of the deactivation of the capsaicin response.

A single pulse protocol was utilized to further investigate the deactivation rate of the capsaicin-activated response. In these experiments a slowing of the deactivation rate of the capsaicin current occurred at a pH level (pH 6.5) that was insufficient to produce an increase in the peak current. This decrease in the deactivation rate resulted in a residual current at the end of the protocol that subsequently deactivated when the external solution was returned to pH 7.4 with a time course similar to control capsaicin responses (Figure 8A). Reducing the pH to 4.0 further slowed the deactivation rate of the capsaicin-evoked current. Both an increase in the peak current and a final deactivation with biphasic kinetics predominated by the fast deactivating acid responses are observed (Figure 8B).

### Effect of co-application of acidic pH and capsaicin on the apparent affinity of a competitive TRPV1 antagonist

**Figure 9:**
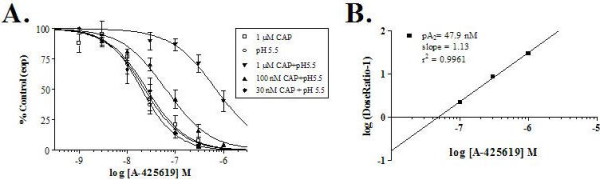
**Potency of A-425619 is altered by co-application of capsaicin and acidic pH**. A) A-425619 inhibits currents activated by half maximal concentrations of either capsaicin (1 μM) or protons (pH 5.5) with similar efficacy (20.1 vs. 25.9 nM). Co-application of these same concentrations of capsaicin and acidic pH results in a significant rightward shift in the IC_50 _of A-425619 (704 nM). Reducing the capsaicin concentration to 100 nM (near the EC_75 _under acidic conditions) or 30 nM (~EC_35_) shifts the IC_50 _of A-425619 back to the left (63.6 and 23.2 nM respectively). B) Schild plot of the effect of A-425619 at different capsaicin concentrations. The resulting data points were fit with a linear regression with a slope of 1.13 and a pA_2 _value of 47.9 nM. The linear relationship is consistent with the results that show acidic conditions increases the affinity of capsaicin for the TRPV1 channel and that A-425619 is a competitive antagonist at the capsaicin site.

A-425619 is a highly potent TRPV1 antagonist that is competitive at the capsaicin binding site [15]. Since acidic pH was found to increase the apparent affinity of capsaicin for TRPV1 by both increasing the activation rate and decreasing the deactivation rate of capsaicin-activated currents we evaluated the potency of A-425619 under various conditions. Inhibitory concentration response curves were generated for A-425619 (0.1–1000 nM) against TRPV1 channel currents activated by acid and capsaicin alone, as well as in combination. Normalized average peak currents evoked by the different activators were plotted as a function of A-425619 concentration and fitted with a logistic equation (Figure 9). Currents that were activated by the ~EC_50 _concentration of capsaicin (1 μM) or acid (pH 5.5) alone were inhibited with similar potency (IC_50 _= 25.9 nM, n = 6 and IC_50 _= 20.1 nM, n = 8 respectively). However, co-application of 1 μM CAP + pH 5.5, concentrations which produce near maximal response when applied together, produced a significant rightward shift in the IC_50 _of A-425619 to 704 nM (n = 3). In comparison, the potency of A-425619 on currents activated by co-application of 100 nM capsaicin + pH 5.5, which represents a response near the capsaicin EC_75 _under these acidic conditions, was 63.6 nM under these conditions (n = 4). By further reducing the capsaicin concentration to 30 nM (~EC_35 _under acidic conditions), the IC_50 _for A-425619 is 23.2 nM (n = 4). Data from the inhibitory concentration response curves for A-425619 were converted to produce a three point Schild plot. The shift in the IC_50 _of A-425619 (Figure 9B) at different concentrations of capsaicin was fit with a linear regression with a slope of 1.13 and a pA_2 _of 47.9 nM. These results are consistent with competitive mechanism of action for this antagonist (Table 3).

**Table 3:** Comparison of the antagonist effects of A-425619 at TRPV1 responses evoked by different agonist(s). Values are the calculated IC_50_'s and Hill slopes derived from fits of average normalized responses following the concentration-dependent inhibition by the TRPV1 competitive antagonist A-425619.

**ACTIVATOR**	**IC_50 _**(nM)	**Hill Slope**
**1 μM Capsaicin**	20.1	-1.1
**pH 5.5**	25.9	-1.2
**30 nM CAP + pH 5.5**	23.2	-1.1
**100 nM CAP + pH 5.5**	63.6	-0.95
**1 μM CAP + pH 5.5**	704.0	-0.93

## Discussion

Recent evidence has illustrated the importance of TRPV1 channels as key integrators of painful stimuli. These receptors may be an important target for the development of novel analgesics. TRPV1 channels are directly activated by acid, heat and endogenous ligands such as NADA, OLDA and anandamide. In addition, inflammatory mediators such as bradykinin have been shown to sensitize TRPV1 through activation of protein kinases [16]. The sensitized TRPV1 channel has heightened responses, and, under these conditions, activators bind the channel with higher affinity. For instance, TRPV1 is normally activated at temperatures greater than 42°C, but after the channel has been sensitized, temperatures <35°C can result in channel activation [17]. Therefore, TRPV1 is not only responsive to multiple painful stimuli, but the additive or synergistic interactions between multiple stimuli make the receptor well suited as an integrator and coincidence detector of painful stimuli and thus underscore its potential as a significant contributor to nociception and hyperalgesia.

In this study we investigated the interaction of acid and capsaicin at TRPV1 in order to understand the mechanism by which these two activators modulate the effects of each other. We found that increasing the concentration of capsaicin or protons resulted in changes in the activation and deactivation rate of the activated currents. In addition, acid responses deactivated about 10-fold faster than capsaicin responses. These differences in deactivation rates may be due in part to the proposed different interaction sites of these two activators with the channel, i.e., the acid site has been proposed to be extracellular while capsaicin site is predominantly intracellular [18]. Alternatively, the different deactivation rates may be the result of differences in the binding affinity of the activators to the channel.

It has previously been shown that acidic conditions can alter the TRPV1 affinity for capsaicin [14] and that mildly acidic conditions that cannot directly activate the channel can alter capsaicin responses [11]. On a single channel level, acidic pH has been shown to potentiate capsaicin binding to TRPV1 as well as increase channel gating [10]. In the present study, we have confirmed these results and demonstrated the novel findings that these changes are due to a slowing of the deactivation rate and an increase in the activation rate of capsaicin-activated currents at acidic pH. In combination, these changes in kinetics result in larger current amplitudes due to a higher probability of channels being bound by agonist. These current amplitudes are similar to what is observed at higher capsaicin concentrations at neutral pH. Thus, one would expect that under acidic conditions 1 μM capsaicin would produce a similar response at TRPV1 as 10 μM capsaicin does at physiological pH, consistent with the 10-fold increase in the apparent affinity for capsaicin at acidic pH.

In contrast to the increased activation rate, the deactivation rate of capsaicin-activated currents under acidic conditions was considerably slower than the rate observed with high capsaicin concentrations at pH 7.4 (see Figure 1 and 5). It is possible that binding of protons alters the conformation of the channel in a way that traps capsaicin so it cannot be released unless the proton interaction is terminated. A recent publication has shown that acidic conditions, in combination with agonists, lock the TWIK-2 channel in an open conformation that persists even after the pH is neutralized [19]. The data in our study suggest that occupancy of the proton binding site of TRPV1 might result in an allosteric change in the receptor that prevents the release of capsaicin. However, a return to physiological pH could rapidly remove this trap and allows capsaicin to be released. The relatively fast deactivation rate of the acid response would provide opportunities for capsaicin to be released, although prolonged exposure to high concentrations of protons may dramatically reduce this probability. Further studies and kinetic modeling are needed to determine if this hypothesis can be supported.

Since TRPV1 can be activated by a variety of stimuli and these stimuli can cross-sensitize the receptor to each other, TRPV1 exhibits complex pharmacology. Cross-sensitization of the channel could potentially result in altered potency and/or efficacy of TRPV1 antagonists. Indeed, we have shown in these electrophysiological studies that application of capsaicin at acidic pH can significantly decrease the *in vitro *potency of A-425619, a competitive antagonist at the capsaicin-binding site of TRPV1, and that this shift becomes particularly dramatic under conditions at or approaching maximal channel activation. In contrast, A-425619 has similar potency (~20 nM) for blocking TRPV1 channel responses to half maximal concentrations of capsaicin or acid. It is possible that binding of the antagonist to the channel results in a conformational change that inhibits channel function independent of the mode of activation. Although there are multiple mechanisms for channel activation, a single site for antagonists may exist that is sufficient to prevent or modulate channel opening. This site most likely is the same or overlaps significantly with the site for capsaicin binding, since A-425619 is competitive with capsaicin [15].

## Conclusion

We report the novel finding that acid alters both the activation and deactivation rates of capsaicin responses and thus provide a mechanism for the increase in potency of capsaicin, and perhaps other agonists, under acidic conditions. Since inflammation is associated with tissue acidosis, these results enhance understanding of the role of TRPV1 in inflammatory pain.

## Methods

### Cell Culture

HEK-293 cells stably expressing the rat TRPV1 receptor were maintained at 37°C in Dulbecco's modified Eagle's medium (DMEM) containing 10% fetal bovine serum. Cells were plated onto glass coverslips coated with polyethyleneimine. Electrophysiological recordings were performed 1–4 days following plating.

### Electrophysiology

HEK-293 cells were maintained at room temperature in an extracellular recording solution (pH 7.4, 325 mOsm) consisting of (in mM): 155 NaCl, 5 KCl, 2 CaCl_2_, 1 MgCl_2_, 10 HEPES, 12 glucose. Patch-pipettes composed of boroscilicate glass (1B150F-3; World Precision Instruments, Inc., Sarasota, FL, USA), were pulled and fire-polished using a DMZ-Universal micropipette puller (Zeitz Instrumente GmbH, Munich, Germany). Pipettes (2–6 MΩ) were filled with an internal solution (pH 7.3, 295 mOsm) consisting of (in mM): 122.5 K-aspartate, 20 KCl, 1 MgCl_2_, 10 EGTA, 5 HEPES, 2 ATP·Mg. For experiments where pH was lowered (6.8 – 4.0) MES was used in place of HEPES in the external solution. No ASIC-like responses were evident in our TRPV1 expressing HEK cells but were recorded in HEK-293 null cells in response to application of acidic pH (unpublished observations). Standard whole-cell recording techniques were utilized for voltage-clamp studies using an Axopatch 200B amplifier (Axon Instruments, Foster City, CA, U.S.A). Coverslips plated with HEK-293 cells were placed in a perfusion chamber and following establishment of whole-cell recording conditions bath perfusion (~2 ml/min) was initiated. Application of control bath solution through a multi-barrel application device with a common 360 μm polyimide tip (Cell Microcontrols, Norfolk, VA, U.S.A), positioned ~100 μm from the cell, was continued throughout the recording except during drug application. Each drug reservoir was connected to solenoid teflon valves that were controlled by a ValveLink16 system (AutoMate Scientific, CA, San Francisco, U.S.A). Drug application protocols were established using pCLAMP (Axon instruments) software that controlled rapid valve switching through the ValveLink system. Drugs were applied by gravity feed through the drug application device for durations described in the results section. Solution exchange times were determined by applying 90% external solution to an open tip electrode positioned at the same distance as during a typical recording. Following a delay of ~50 ms from the opening of the solenoid valve, currents activated at a rate of 72 ± 9 ms with a 10–90% rise time of ~120 ms (n = 4). Similarly, the current deactivated with an deactivation rate of 84 ± 16 ms (n = 4). These values give an approximation of the limitations of the drug exchange for this system across an open tip electrode and indicate that our measurements of activation and deactivation rates of whole cell currents should not be significantly affected by solution exchange times. Prior to and following each drug application external solution was applied through the application device to ensure rapid washout. Each drug application sequence was followed by a washout period of 90 to 120 seconds.

Capsaicin concentration response curves were generated by applying increasing concentrations of capsaicin for 4 seconds followed by a 90 second washout period. These responses were evoked at pH 7.4 and pH 5.5 and the responses were normalized due to large variation in peak amplitude between cells, and the subsequent average data was fitted with a four-parameter logistic equation (see below). To determine potential changes in the efficacy of capsaicin responses a separate experiment was performed comparing peak responses to 30 μM capsaicin at pH 5.5 and pH 7.4 on the same cell. Generally there was substantial rundown of the currents during the first few applications of 30 μM capsaicin. Therefore, comparisons between the responses to pH 5.5 and pH 7.4 were made after two consecutive applications of capsaicin produced similar responses.

Inhibitory concentration response curves were performed by applying the agonist(s) for 4 seconds followed by 8 seconds of co-application of agonist and antagonist. Current levels at the end of the application period were measured and normalized to the current amplitude at the end of a 12 second control application of agonist alone. Normalized responses were then plotted as a function of antagonist concentration and fitted with a four-parameter logistic equation (see below).

Data acquisition and analysis were performed using pCLAMP 9.0 and subsequent graphs were plotted and statistical analysis done using GraphPad Prism (Graphpad Software, San Diego, CA, U.S.A). Activation and deactivation rates were calculated in Clampfit using a single exponential equation provided with the software:



Agonist and antagonist concentration-response curves were fitted by a least-squares regression to the logistic equation provide in the GraphPad software:



A Schild plot for A-425619 was constructed by converting data from inhibitory concentration response curves obtained at different capsaicin concentrations (in the presence of acidic pH) to capsaicin concentration response curves under different concentrations of antagonist. The resulting curves were fit with the logistic equation shown above and the resulting EC_50 _were used to calculate the log dose ratio that was then plotted as a function of capsaicin concentration. A linear regression was then used to fit these data points.

All reagents were obtained from Sigma Chemical Co. (St. Louis, MO, U.S.A.) except A-425619 (synthesized at Abbott Laboratories, Abbott Park, IL, U.S.A).

## Authors' contributions

TRN carried out the electrophysiological studies, conceived of the study, participated in the design of the study, performed the statistical analysis and drafted the manuscript. PH generated the stable TRPV1 cell line. MFJ and CSS participated in the design of the study. All authors read and approved the final manuscript.
